# Role of Osteoprotegerin and Its Gene Polymorphisms in the Occurrence of Left Ventricular Hypertrophy in Essential Hypertensive Patients

**DOI:** 10.1097/MD.0000000000000154

**Published:** 2014-12-02

**Authors:** Anna Shen, Xuwei Hou, Deguang Yang, Tingrong Liu, Dezhong Zheng, Liehua Deng, Tao Zhou

**Affiliations:** From the Department of Cardiology, TheThird Affiliated Hospital of Southern Medical University, No.183, West Zhongshan Ave, Tianhe District, Guangzhou (AS, DY, TL, DZ, TZ); Department of Cardiology, Hangzhou Hospital, Nanjing Medical University, 261 huasha Road, Hangzhou (XH); and Department of Critical Care Medicine, Affiliated Hospital of Guangdong Medical College, No. 57 Southern Renmin Avenue, Zhanjiang (LD), Guangdong, China.

## Abstract

The aim of the study was to investigate the role of osteoprotegerin (OPG) in left ventricular hypertrophy (LVH) development in patients with essential hypertension (EH).

A total of 1092 patients diagnosed with EH were recruited. The LVHs were determined and OPG gene polymorphisms were genotyped.

Patients with LVH had a significantly higher mean serum OPG level than those without LVH. The 1181CC genotype carriers had significantly lower risk for LVH compared with GC and GG genotype carriers. The serum OPG level and OPG 1181 G>C polymorphism were found to be independent risk factors for the occurrence of LVH in hypertensive patients. In vitro study shows that OPG overexpression upregulates cell surface size, protein synthesis per cell, and hypertrophy- and fibrosis-related proteins in both cardiomyocytes and cardiac fibroblasts, whereas OPG inhibition can abolish the above-mentioned changes. Consistent with the in vitro data, our in vivo study revealed that the OPG administration induced the LVH in hypertensive rats.

This study is the first to report the close association between OPG and LVH development in EH patients and the regulatory effect of OPG on cardiomyocytes and cardiac fibroblasts.

## INTRODUCTION

High blood pressure is associated with adverse morphological and functional changes in the cardiovascular system, including left ventricular hypertrophy (LVH)^1,2^ Epidemiological studies show that the prevalence of LVH in Chinese patients with essential hypertension (EH) was about 25% to 35%.^3^ LVH is regarded as an independent risk factor for cardiovascular morbidity and mortality.^4–7^ Many factors, that is, blood pressure level, duration of hypertension, age, obesity, diet, and pharmacologic treatment may influence the occurrence and degree of LVH.^8,9^ In addition, increasing evidence show that the genetic factors are related to LVH as well.^10–12^ A number of candidate genes responsible for LVH have been studied in different ethnic populations, but the results of these studies are inconsistent and often controversial.^13–15^

Osteoprotegerin (OPG) is a member of the tumor necrosis factor receptor superfamily of cytokines and a soluble receptor for the receptor activator for nuclear factor-κB ligand. Serum OPG is involved in the vascular remodeling and dysfunction in type 1 and type 2 diabetes mellitus (DM).^16^ The association of serum OPG with left ventricular mass was reported in African-American adults with hypertension and in the general population.^17,18^

The human *OPG* gene located on chromosome 8 represents a single-copy gene with 5 exons spanning 29 kb of the human genome. The promoter region of the human OPG gene contains various binding sites that are able to mediate the stimulation of *OPG* gene expression.^19^ Several polymorphisms at the OPG promoter have been reported to be associated with vascular morphology and function in different populations.^20,21^ However, the role of OPG gene polymorphisms and serum OPG level in the development of cardiac remodeling, such as LVH development under hypertensive condition, has not been addressed. In the present study, we enrolled patients with EH to investigate the possible association between OPG genetic polymorphisms, serum OPG level, and LVH occurrence.

## METHODS

### Enrollment

This is a hospital-based case–control study. A total of 1092 patients diagnosed with EH were recruited in our hospital from April 2007 to September 2011. According to the presence or absence of LVH, subjects were divided into LVH+ (EH with LVH) and LVH− (EH without LVH) groups. To avoid the influence of drug therapy on LVH, the enrollment was matched in the LVH+ and LVH− groups according to the baseline antihypertensive agents and therapy duration. The baseline therapy includes the angiotension-converting enzyme inhibitors, calcium-channel blocker, angiotension II type 1 receptor blocker, β receptor blocker, and diuretics. The study protocol was approved by the ethics committee of Southern Medical University. All subjects provided written, informed consent in compliance with the Code of Ethics of the World Medical Association (Declaration of Helsinki).

### Determination of Biochemical Variables and Clinical Data

Blood samples were collected after a 12-hour overnight fast. The serum creatinine,^22^ total cholesterol, total triglyceride (TG), high-density lipoprotein cholesterol (HDL-C), low-density lipoprotein cholesterol (LDL-C), and blood glucose were acquired. High-sensitivity C-reactive protein measurements were performed using a commercially available high-sensitivity assay (Roche Diagnostics, Branchburg, NJ). In addition, a complete medical history was obtained from all subjects, including DM, alcohol intake, cigarette smoking, weight, height, body mass index (BMI), systolic blood pressure (SBP), and diastolic blood pressure (DBP).

### Measurement of LVH

All subjects underwent echocardiography using a Hewlett–Packard imaging system (Sonos 2500 model, Palo Alto, CA). All measurements were performed in 3 cardiac cycles at end-diastole and end-systole, by the 2 investiga1tors who were blind to the genotypes of the patients. Left ventricular (LV) mass (LVM) was calculated at end-diastole using the formula: 0.8 × 1.04[(IVSd + LVIDD + PWTd) − LVIDD] + 0.6 (IVSd, interventricular septal thickness; PWTd, posterior wall thickness; LVIDD, LV end-diastolic internal dimension), which yields values closely related (*R* = 0.90) to necropsy LV weight. LVM was divided by height 2.7 m to obtain LVM index (LVM index). LVH was defined as LVMI >49.2 g/2.7 m for men and >46.7 g/2.7 m for women.^23^

### Serum OPG Detection

Serum OPG was quantified by an enzyme immunoassay using commercially available matched antibodies (R&D Systems, Minneapolis, MN). The intra-assay and interassay coefficients of variation were 3.6% and 10.6%, respectively.^24^

### OPG Genotyping

The genomic DNA was extracted from the peripheral blood leukocytes using a QIAamp DNA blood kit (Qiagen GmbH, Hilden, Germany). The genomic DNA containing the 209 G > A polymorphic portion in the OPG promoter was amplified by polymerase chain reaction (PCR), as described.^25^ The 163 A → G, 245 T > G and 1181 G > C polymorphisms were analyzed by amplifying the specific DNA fragments by PCR using the oligonucleotide primers and PCR reaction steps described elsewhere.^26^ The PCR products were digested with AseI, TaqI, HinfI, and SmlI restriction endonuclease to detect the 163 A > G, 245 T > G, and 1181 G > C polymorphisms, respectively, and electrophoresed through agarose gels containing ethidium bromide. The gels were visualized under ultraviolet light and photographed.

### Rat Cardiomyocytes and Cardiac Fibroblast Culture

The whole hearts from neonate rats were isolated, minced, and rinsed in hood by using a device invented by Xuwei Hou (patent number: CN 101955884 A and CN 101955884 B). Collagenase (Sigma Aldrich, Bornem, Belgium, 95 U/mL) was used for digestion. Obtained cell population was used for isolation of fibroblasts and cardiac cells by immunomagnetic cell sorting based on Miltenyi Biotec (Bergisch Gladbach, Germany) protocol. Fibroblasts were purified by positive selection with antifibroblast MicroBeads and passage through MS columns placed in magnetic field, followed by incubation of the collected negative fraction with anti-human-CD117 MicroBeads and positive selection of CD117-positive cardiac cells.^27^

### OPG Inhibition and Overexpression

The adeno-associated virus (AAV)-mediated OPG plasmid was a gift from XH from Dalian Medical University, China. Cultured cells were transfected with OPG siRNA (Ambion Inc, Woodward Austin, TX) and control siRNA (Ambion, Invitrogen, Austin, TX) using lipofectamine, Invitrogen,Grand Island, NY for 4 to 6 hours.^28^

### Hypertrophy Assays

Cardiomyocytes and cardiac fibroblast were stimulated with 10(−6) mol/L angiotensin (Ang) II for 48 hours. To examine changes in cell morphology and cytoskeleton, cells were fixed in 4%paraformaldehyde, stained with fluorescein isothiocyanate-conjugated Phalloidin (Sigma-Aldrich, St. Louis, MO) for 30 min and mounted in Vectashield with 4′,6-diamidino-2-phenylindole (Vector Laboratories, Peterborough, UK). Cellular hypertrophy was evaluated by measuring cardiomyocytes and cardiac fibroblasts surfaces using a digital image analysis system (Leica QwinV3, Leica Microsystems Ltd, Cambridge, UK). Five random fields (with approximately 10–15 cells per field) from every sample were averaged and expressed as μm^2^/cell. All experiments were repeated 3 times.

### Measurement of Protein Synthesis in Cardiomyocytes and Cardiac Fibroblast

The AngII-treated cells were trypsinized and counted using a cell counting chamber (Beckman Coulter, Fullerton, CA) and then lysed. The cell lysates were prepared to determine protein content by Bradford protein assay. Then the protein synthesis of cells was determined by dividing the total amount of protein by the number of cells, namely, protein per cell.^29^

### Western Blotting Analysis for Cardiomyocyte Hypertrophy and Fibrosis Markers

Collected cardiomyocytes were separated by trypsin and the protein concentration in the supernatant was determined with a BCA protein assay kit (Beyotime, Jiangsu, China). The isolated protein (25 μg) from cardiomyocytes was separated by 10% SDS Polyacrylamide Gel Electrophoresis and transferred onto polyvinylidene difluoride nylon membranes. The blots were probed with mouse anti-rat atrial natriuretic peptide,^30^ troponin I, α-myosin heavy chain (α-MHC), collagen type I, III, metalloprotein-2 (MMP-2), MMP-9, and transforming growth factor (TGF)-β1 (all 1: 1000 dilution, Millipore Corporation, Billerica, MA), and anti-GAPDH antibody (1: 1000 dilution; Santa Cruz Biotechnology, Santa Cruz, CA). Then the membranes were incubated with horseradish peroxidaseconjugated secondary antibodies (1:1000 dilution), and visualized using an ECL detection kit (Amersham Biosciences,Piscataway, NJ). The optical densities of the bands were quantified by densitometric analysis performed with a quantitative imaging system (Bio-Rad Laboratories, Hercules, CA). All western blot experiments were repeated 3 times.

### Animal Preparation

The spontaneously hypertensive rats (SHR) were used to investigate the effect of OPG on LVH in vivo. All procedures were carried out in accordance with conventional guidelines for experimentation with animals. Fourteen 14-week-old SHR males were randomly divided into a SHR + OPG group (n = 7) and a SHR control group (n = 7). The rats from SHR + OPG group were given OPG (5 mg/kg/day) via lateral vein tail injection for 7 days, whereas the SHR control group was given only saline at the same volume. Systolic and diastolic blood pressures (SBP and DBP) were measured with an automated multichannel system, using the tail-cuff method with a photoelectric sensor.

### Left Ventricular Hypertrophy

Rats were anesthetized (pentobarbital, 60 mg/kg, i.p.) 4 weeks after OPG injection. The heart was removed from the body immediately after sacrifices. The atrium was removed, and all the epicardial fat was scraped off. The right and the left ventricles were separated, regarding the interventricular septum as an integral part of the LV, and this portion was weighed. LVH was calculated by using left ventricular weight/body weight ratio (LVW/BW ratio).^31^

### Statistical Analysis

Data on quantitative characteristics are expressed as means ± standard deviation or mean. Data on qualitative characteristics are expressed as percent values or absolute numbers, as indicated. Differences in demographic characteristics and vascular risk factors between patients and controls were compared by using Student *t* test for continuous variables and the *χ*^2^ test for all categorical variables. Tests for Hardy–Weinberg equilibrium for the gene polymorphisms were conducted using *χ*^2^ tests. Genotypes and allele frequencies were compared by *χ*^2^ analysis. Multivariate logistic regression analysis was used to determine the influence of OPG polymorphisms on LVH risk, with controlling potential confounding risk variables. A forward stepwise (Likelihood Ratio) procedure was used for multivariable analysis. The receiver-operating characteristics (ROC) curves for serum OPG were constructed for discrimination between hypertensive patients with or without LVH. Data were analyzed with the SPSS 13.0 package (SPSS Inc, Chicago, IL) and the results were considered statistically significant at *P* < 0.01 using a 2-tailed test.

## RESULTS

The clinical and biochemical characteristics of all subjects are shown in Table 1. A total of 443 patients were assigned to the LVH+ group and 649 to the LVH− group. There were no significant differences in age, sex, alcohol intake, BMI, TG, Total Cholesterol (TC), HDL, LDL, and sCr between LVH+ and LVH− groups. The mean LVMI in LVH+ group was 51.3 ± 3.8 g/m^2^, whereas that in LVH− group was 43.3 ± 4.1 g/m^2^ (*P* < 0.001, Table 1).

**TABLE 1:**
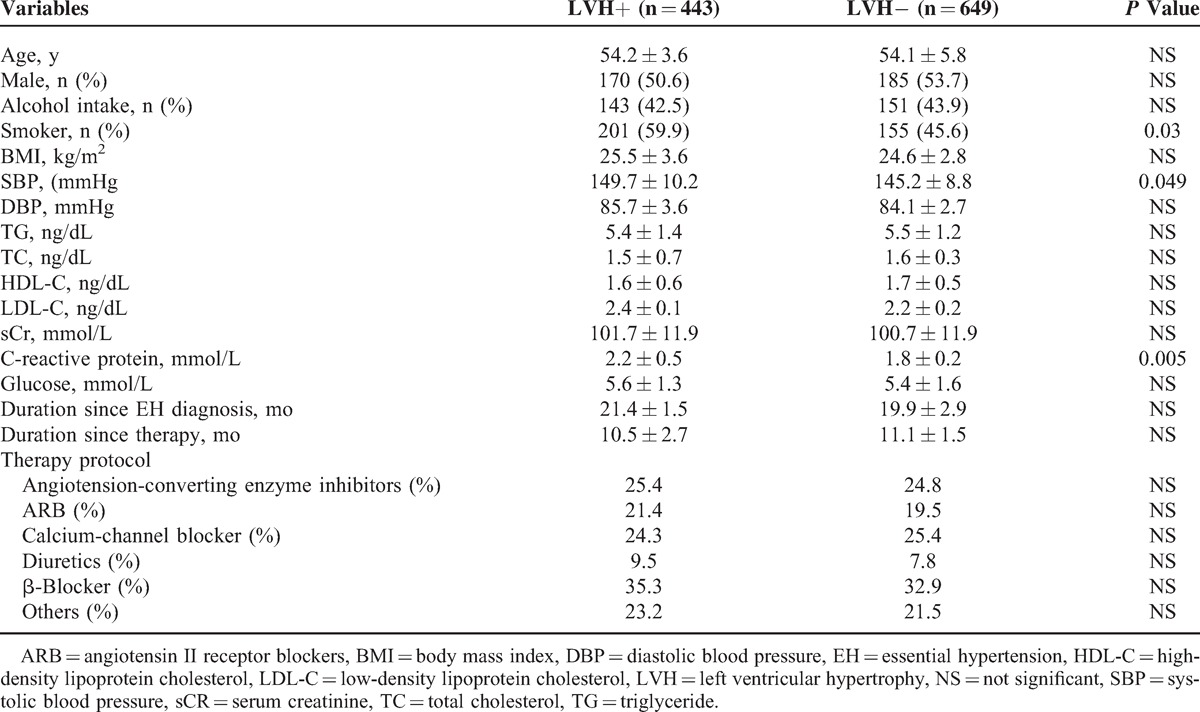
Clinical and Biochemical Characteristics of all Participants

Table 2 shows the distributions of genotypes and the allele frequencies of OPG gene in 2 groups. Genotype frequencies of OPG polymorphisms in LVH+ and LVH− were in Hardy–Weinberg equilibrium (all *P* > 0.05). The genotype and the allele frequencies of 163 A > G and 245 T > G were not significantly different between LVH+ and LVH− groups (all *P* > 0.05). However, the genotypes and allele frequency of 1181 G > C were significantly different between the 2 groups. LVH+ patients had a markedly lower CC genotype than LVH− patients (18.06% vs 31.12%, global *P* < 0.001). With the 1181GG genotype as reference, multivariate logistic regression analysis showed that the 1181CC genotype carriers had a markedly lower chance to develop LVH in our study (adjusted odds ratio [OR] = 0.350, 95% CI 1.30–3.11, adjusted *P* = 0.001) with adjustment for age, sex, alcohol intake, smoking status, BMI, TG, TC, HDL, LDL, sCr, HBP, and DBP levels.

**TABLE 2:**
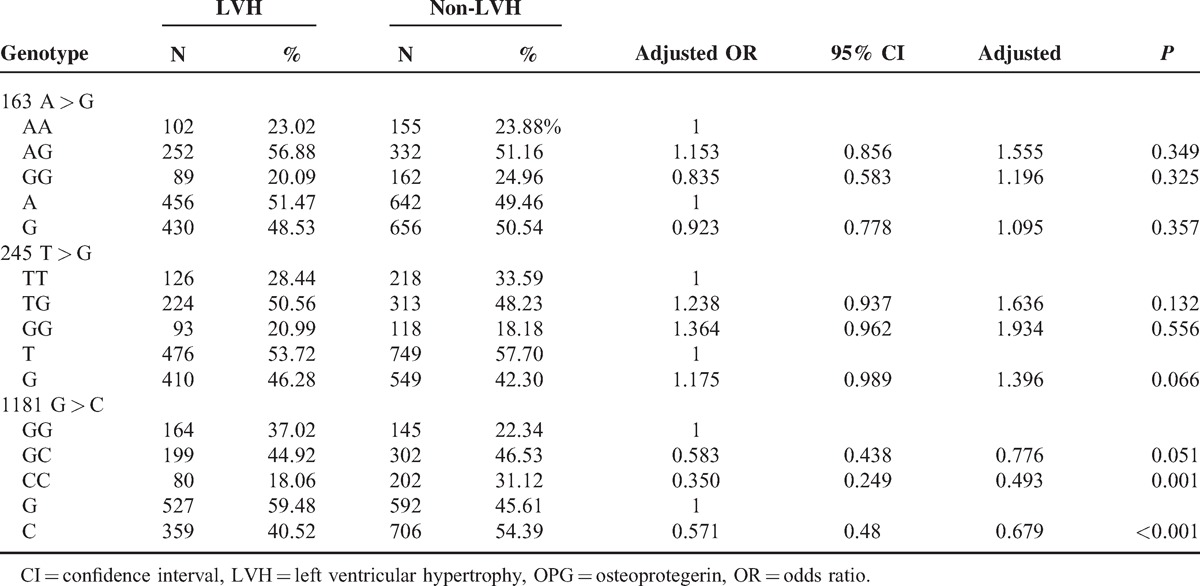
Distributions of Genotypes and the Allele Frequencies of OPG in LVH and non-LVH Patients

**FIGURE 1:**
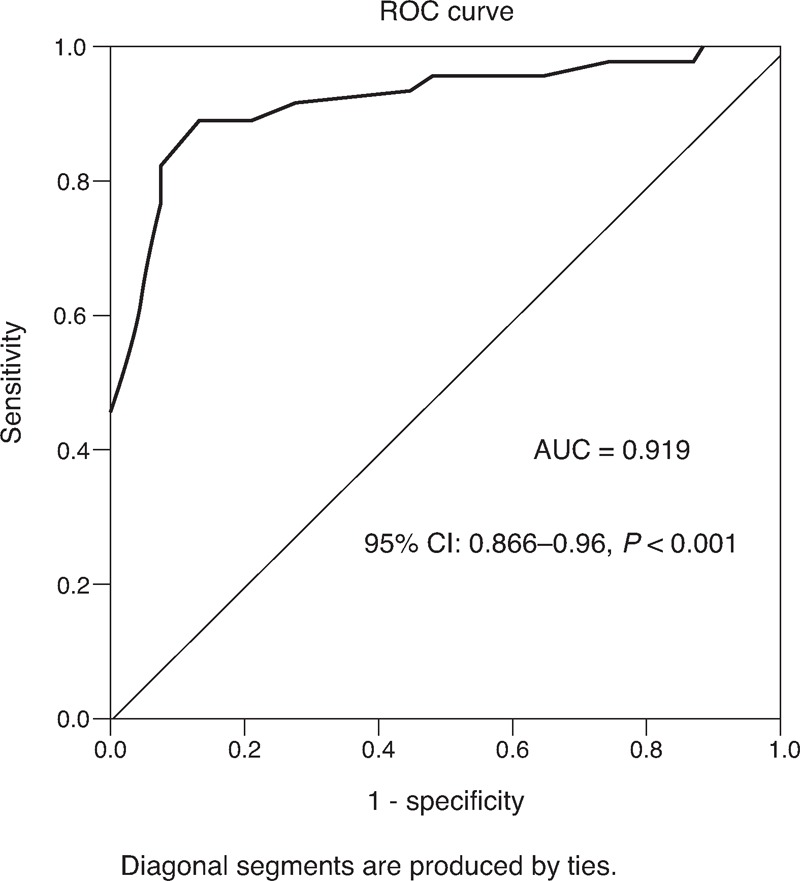
ROC curve of OPG serum level for LVH incidence in EH patients.

We next investigated the serum OPG levels according to the presence or absence of LVH. Serum OPG levels in LVH+ group (3.42 [2.62–4.0] pmol/L) were significantly higher than that in LVH− group (2.54 [1.54–3.17] pmol/L, *P* < 0.001). The ROC curves for serum OPG were constructed for discrimination between hypertensive patients with or without LVH. The area under curve (AUC) for serum OPG is 0.919 (95% CI 0.866–0.96, *P* < 0.001, Figure 1). The association between serum OPG levels and LVH in hypertensive patients was also tested by the multivariate logistic regression model with the dependent variable as hypertension with or without LVH. After adjusted for traditional covariates mentioned above in the multivariate logistic regression analysis, serum OPG levels remained independently associated with LVH in hypertensive patients (adjusted OR = 2.26, adjusted *P* < 0.001).

**FIGURE 2:**
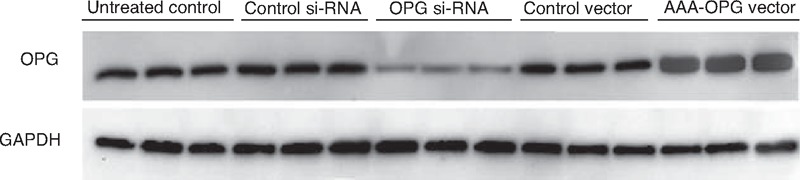
The OPG expressions in cells after OPG inhibition and overexpression. OPG = osteoprotegerin.

In the in vitro study, the OPG inhibition by si-RNA and overexpression by AAV-OPG vector were confirmed by western blot assay (Figure 2). OPG si-RNA transfection dramatically inhibited the OPG expression, whereas AAA-OPG vector increased OPG expression level. The control Si-RNA and control vector transfection had the similar OPG expressions compared with untreated control cells.

**FIGURE 3:**
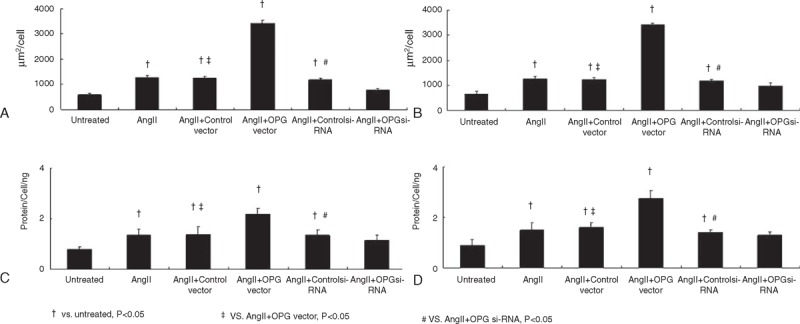
The effect of OPG on cell surface size and protein content per cell in cardiomyoctyes and cardiac fibroblasts. OPG = osteoprotegerin.

Compared with untreated cells, AngII treatment dramatically increased the cellular surface in both cardiomyoctye (Figure 3A) and cardiac fibroblasts (Figure 3B). Compared with cells receiving AngII only, cells receiving AngII+AAA–OPG vector had significant increases of cellular surface, whereas cells with OPG inhibition by si-RNA transfection had markedly decreased cellular surface. Compared with untreated cells, the AngII treatment also markedly increased protein concentrations per cell in both cardiomyoctye (Figure 3C) and cardiac fibroblasts (Figure 3D). Similar to cellular surface changes, the OPG overexpression unregulated the cellular protein synthesis, which is dramatically downregulated by OPG si-RNA transfection.

**FIGURE 4:**
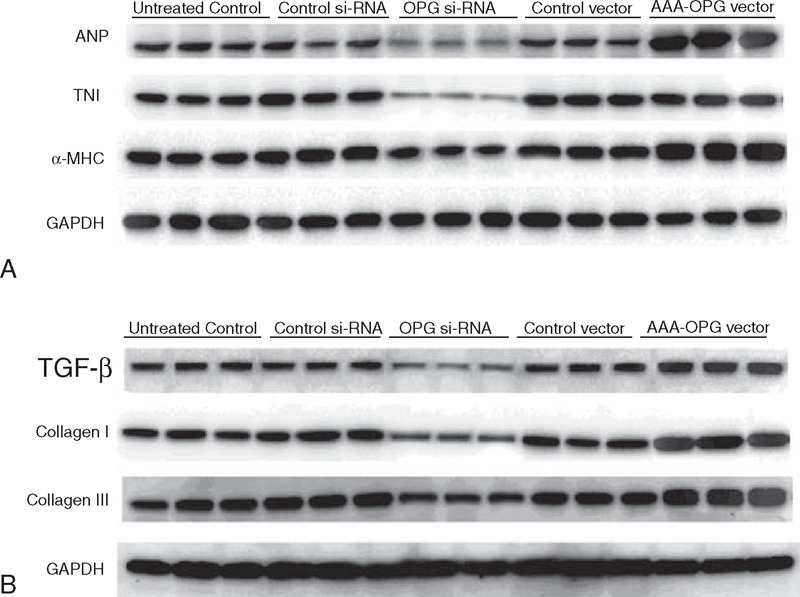
The effect of OPG on the hypertrophy and fibrosis-related protein expressions in cardiomyoctyes and cardiac fibroblasts. OPG = osteoprotegerin.

We next analyzed the hypertrophy and fibrosis-related protein expressions by western blot assay. Our results showed that the cardiomyocyte with AAA-OPG overexpression had a dramatic increase in Atrial Natriuretic Peptide (ANP), α-MHC, and troponin I expression compared with cells transfected with control vector. In contrast, the OPG si-RNA transfection to cells induced considerable reduction of the above-mentioned proteins in cardiomyocytes (Figure 4A). In the cardiac fibroblast, AngII treatment increased the fibrosis-related protein expressions, e.g. TGF-b1,collagen I and III than untreated cells. OPG overexpression further increased these proteins’ expression levels in Ang-pretreated cells, whereas OPG si-RNA transfection inhibited their expression levels (Figure 4B).

In our in vivo study, we did not find the OPG injection significantly affecting the SBP and DBP level and body weight in our SHR models. However, rats in the SHR + OPG group had a significantly higher LVW and LVW/BW ratio than SHR control group (both *P* < 0.01, Table 3).

**TABLE 3:**
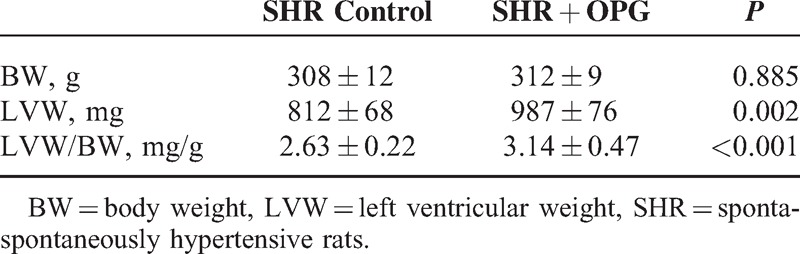
BW, LVW, and LVW/BW Ratio Between Rats From the SHR Control and SHR + OPG Groups

## DISCUSSION

In the present study, we explored the role of OPG gene polymorphisms and serum OPG level in the occurrence of LVH in Chinese EH patients. We found that only 1181 G > C was associated with the serum OPG level and LVH risk in EH patients. The LVH+ patients had a significantly higher serum OPG levels than LVH− patients. The serum OPG levels, as well as the 1181 G > C were found to be independent risk factors for the occurrence of LVH in hypertensive patients. In vitro study shows that OPG overexpressoin can upregulate the cell surface size, protein synthesis per cell, and hypertrophy and fibrosis-related proteins in both cardiomyocytes and cardiac fibroblasts, whereas OPG inhibition can abolish the above-mentioned changes. Consistent with the in vitro data, our in vivo study also revealed that the OPG administration induced the LVH in hypertensive rats. This study, to the best of our knowledge, is the first to report the close association between OPG and LVH development in EH patients and the regulatory effect of OPG on cardiomyocytes and cardiac fibroblasts.

OPG is inflammatory cytokine traditionally linked to the regulation of bone remodeling. Interestingly, recent studies have linked OPG to cardiovascular diseases.^32^ For example, plasma OPG has been shown to reflect the severity of coronary artery disease at angiography and to predict the incidence of and mortality from cardiovascular diseases in the community.^33^ Enhanced myocardial protein levels of OPG in both experimental and clinical heart failure were detected in human patients.^34^ Another recent study revealed that OPG may be involved in cardiac remodeling in immunoinflammatory myocardial diseases and progression of chronic heart failure and thus may represent targets for intervention in this disorder.^33^ Elevated OPG levels are significantly associated with endothelial dysfunction in type 2 DM, suggesting that OPG may act as an important regulator in the development of vascular dysfunction in DM.^16^ In this study, we found that the OPG serum level is significantly associated with the incidence of LVH in EH patients. This finding provides more information to the role of OPG played in cardiovascular disease, suggesting that the serum may be used as a biological marker for the LVH development in EH patients.

Genetically, the 163 A > G (rs3102735) is located in promoter, 1181 G > C (rs2073618) located in exon I, and 245 T > G (rs3134070) is located in promoter of the OPG (chromosomal location 8q24).^35^ Of all genetic variants, the polymorphism at 1181 G > C locus seems to be more associated with cardiovascular disease than the others. A recent study in Polish population revealed carriers of the homozygous CC of 1181 *OPG* gene were shown to have normal coronary arteries more frequently when compared with heterozygotes for CG or homozygotes for GG, but not 209 C/T and 245 C/T C polymorphisms.^36^ However, another study in Korean population failed to show a significant association between OPG and RANK polymorphisms and acute coronary syndrome occurrence.^37^ In Chinese EH patients, we found that the polymorphism at 1181 G > C locus is closely associated with the risk and the severity of for LVH development.

To date, there is no study reporting the direct effect of OPG on the hypertrophic response of cardiomyocytes. A recent study reported that osteoprotegerin induces the proliferation of rodent vascular smooth muscle cells both in vitro and in vivo.^38^ We used the rat to harvest the cardiomyocytes and cardiac fibroblast. We found that OPG overexpression can upregulate the cell surface size and protein synthesis per cell, suggesting a direct hypertrophic response of cardiomyocyte under OPG treatment. Meanwhile, OPG also affects the cardiac fibroblast directly. Our previous study shows that osteopontin increased the protein content per cell, the cardiomyocyte surface size, and the expression level of ANP in a dose-dependent manner.^39^ However, it should be noted that in cultured human vascular smooth muscle cells, OPG expression does not affect the proliferation rate and does not change the vascular calcification gene expression patterns.^28^ Thus the effect of OPG on human cardiomyocyte should be further studied.

Several limitations should be addressed in this study. First, this is a single-center-based study and all the participants were ethnically Chinese in southeastern China area. The sample size is relatively small. Second, we do not have human-derived cardiomyocytes for in vitro study. So our in vitro data from rat cardiomyocytes need to be replicated in human cardiomyocytes.
